# Systematic manipulation of experimenters' non-verbal behaviors for the investigation of pain reports and placebo effects

**DOI:** 10.3389/fpsyg.2023.1248127

**Published:** 2023-10-31

**Authors:** Hojjat Daniali, Mollie A. Ruben, Magne Arve Flaten

**Affiliations:** ^1^Department of Psychology, Norwegian University of Science and Technology, Trondheim, Norway; ^2^Department of Psychology, University of Rhode Island, Kingston, RI, United States

**Keywords:** nonverbal behaviors, impression formation, stimuli development, placebo effects, subtle contextual factors, pain reports

## Abstract

**Objective:**

Non-verbal behaviors (NBs) of caregivers affect pain reports and placebo effects. However, little experimental research has systematically examined the caregivers' NBs. This study protocol and preparatory study report a systematic manipulation of experimenters' NBs to investigate pain report and placebo effects.

**Methods:**

We propose an experiment in which videotaped experimenters (VEs) conduct a pain stimulation and a placebo treatment study. The VEs express one positively enhanced NB and keep the other NBs neutral. Participants will be randomized to either the positive facial expressions (+FE), tone of voice (+TV), body movement (+BM), or neutral NBs (i.e., neutral condition; NC) of the VEs. As a preparatory study for proof of concept, two groups of NB coders from Norway and the USA separately rated the degree of NBs (eye contact, body postures and movements, and tone of voice), and impressions of dominance and being in charge, positivity, and expressivity from each NB video. The NB videos had construct validity and reliability. The +BM and +FE were rated as more dominant and in charge than the +TV and the NC. The +FE and +BM were rated as the most positive and expressive NBs, respectively.

**Expected results:**

+FE will have the largest placebo effects on pain and stress levels. However, transmitting the NBs to patients by VEs is challenging. Moreover, controlling for the effects of research assistants present in the testing room is challenging.

**Discussion:**

We propose that caregivers' NBs affect pain reports and placebo effects. Moreover, different NBs elicit different impressions, and a better understanding of the role of caregiver NBs requires more rigorous investigations. Lastly, aiming to investigate the caregiver NBs, the varying degrees of micro-NBs and their effects on the formation of impressions should be considered.

## Introduction

Placebo analgesia, the reduction in pain due to the administration of a medically inactive element (Amanzio and Benedetti, 1999; Vambheim et al., 2021), is caused by positive expectations about a treatment, which is thought to be partly due to the treatment setting, indicating that an effective treatment has been administered (e.g., Miller and Kaptchuk, 2008). Placebo effects are embedded in all kinds of treatments and placebo-controlled trials (Benedetti et al., 2014). Verbal information modulates treatment expectations (Flaten et al., 2006); however, verbal suggestion is just one source of information for patients, and contextual factors, such as the clinic, the doctor–patient relationship, and the caregiver's gender (Levine and De Simone, 1991) and their characteristics (Kállai et al., 2004; Howe et al., 2017), contribute to treatment outcomes and placebo effects (Daniali and Flaten, 2019).

Among these, the non-verbal behaviors (NBs) of caregivers are shown to influence treatment outcome and placebo effects (Benedetti et al., 2014; Czerniak et al., 2016; Daniali and Flaten, 2019). A considerable amount of emotions, thoughts, and feelings are conveyed by NBs (DePaulo and Friedman, 1998), and our NBs can align or contradict with our verbal messages (e.g., Ekman, 1965; Jacob et al., 2013). Even the absence of NBs (i.e., neutral expressions) may convey an impression (e.g., negativity or disengagement) (Said et al., 2009; Knapp et al., 2013).

NBs can be categorized into macro-level and micro-level. Macro-level NBs, also called impressions, are the result of a combination of a series of micro-level NBs (Ambady et al., 2000; Matsumoto, 2006a,b; Burgoon et al., 2011). Micro-level NBs are specific NBs such as smiling, direction of gaze, and limb movements that can generate macro-level NBs (Ambady et al., 2000).

Both micro- and macro-level NBs can be either positively or negatively valenced. Positive NBs are more immediate and reduce the actual or psychological distance between two interactants (Mehrabian, 1969), and negative NBs imply a negative feeling or attitude or increase the actual or psychological distance between the two interactants. Such positivity and negativity in NBs are typically compared against the neutral NBs, which reflect neither positive nor negatively valenced feelings or attitudes.

Patients draw impressions from their caregivers' NBs (e.g., how friendly, competent, warm, empathic, positive, etc. the doctor is), which in turn impact the treatment outcomes. Kraft-Todd et al. (2017) showed that when caregivers' photographs displayed enhanced eye contact, leaning forward, and smiling, participants rated higher impressions of empathy and warmth compared to when the counterpart NBs were displayed to participants. A review by Daniali and Flaten (2019) showed that several positive NBs (e.g., smiling, enhanced eye contact, positive tone of voice, and close proximity to the patient) of caregivers lowered clinical and experimental pain and increased placebo effects, and conversely, negative NBs (e.g., lack of smile, no eye contact, etc.) increased pain and nocebo effects. In addition, Czerniak et al. (2016) also showed that when the clinician displayed positive body postures, escorted the patients, had longer eye contact, and smiled more, the patients could withstand cold pain longer. The positive NBs of the caregivers have also been shown to reduce negative emotions (e.g., van Osch et al., 2017).

However, one issue still not thoroughly investigated is which NBs have the most salient effect on pain reports and placebo effects. To reliably investigate the caregivers' NBs, the NBs must be defined and measured in a systematic and replicable manner. This is a challenging task, as there is no NB dictionary to define and measure NBs (Blanch-Hartigan et al., 2018). This is partially why in most of the available literature, an unspecified group of NBs has been simultaneously manipulated, making the results conflated and unable to show the role of specific NBs (e.g., Kaptchuk et al., 2008).

## Experiment outlook

To answer this, we designed an experiment in which the NBs of the experimenters were separately enhanced, recorded, and then tested on pain reports and placebo effects. A videotaped experimenter (VE) guided a pain experiment and introduced a treatment (a placebo) while expressing specific NBs. Four NB conditions were developed. In three conditions, one NB was positively enhanced, and in the fourth condition, all NBs were neutral. The conditions were positive facial expressions (+FE), tone of voice (+TV), body movements (+BM), and neutral conditions (NCs). This article describes the experiment protocol and the procedures to design and validate the NBs of VEs.

## Experiment protocol

General aims were as follows: (1) Positively enhanced NBs will have a larger reduction in pain and placebo amplitude compared to the NC, (2) the +FE will have the largest reduction in pain and placebo effect compared to other NB groups, (3) larger reduction in pain will be associated with lower stress levels (both physiological and subjective) and (4) the +FE will have the lowest stress level compared to other NB groups.

## Methods

### Participants

Eighty healthy volunteers (40 females and 40 males) between the ages of 18 and 45 will be recruited. The age was limited to a younger age range, as aging might affect pain sensitivity (Yezierski, 2012). Participants will be randomly assigned to four groups (20 participants each group), each with 10 males and 10 females. Each participant will be paid 200 NOK (approximately 20 USD). A history of severe psychiatric disorder, chronic pain, injuries or scars on the right arm, pregnancy, or use of prescription drugs (except birth control pills) will result in exclusion. Participants will be requested not to drink alcohol 24 h prior to the experiment and abstain from large meals, nicotine, caffeine, or energy drinks 3 h prior to the experiment.

### Questionnaires

*Pain intensity and pain unpleasantness* will be rated on an 11-digit numeric rating scale (NRS) as described by Price et al. (1983).

*Stress and arousal* are assessed before and after the stimulations, by the Short Adjective Check List (SACL; Mackay et al., 1978): two adjective pairs, each rated on a scale from “0” to “10.” Stress is measured by asking participants to rate how “relaxed” or “tense,” or how “calm” or “nervous” they are, on a scale from “0” to “10.” Arousal will be assessed the same way on the scales of “sleepy” or “awake,” and “tired” or “energetic.” SACL has been used in several similar studies (Lyby et al., 2010; Bjørkedal and Flaten, 2011; Vambheim et al., 2021).

*CARE*. Three items (items 1, 6, and 8) from the 10-item Consultation and Relational Empathy (CARE) (Crosta Ahlforn et al., 2017) are selected to assess the satisfaction of the VEs.

*Expected efficacy*. After receiving the placebo cream and before the pain stimulation in the conditioning and post-test phases (see Procedure), participants will be asked to rate how much they expect the cream will reduce their pain intensity on an 11-digit NRS.

*Prior experience* with the cream. Participants will be asked whether they have had any former experience with thermal pain-relieving creams. If the answer is yes, they will be asked to rate the efficacy of the cream on an NRS (Aslaksen and Flaten, 2008).

*The Big Five Inventory-Short Form (BFI-10)* (John et al., 1991) and the *Fear of Pain Questionnaire III (FPQ III)* (McNeil and Rainwater, 1998) will be filled out before the experiment begins.

### Psychophysiological measurements

Cardiac activity will be recorded using the MP150 BIOPAC system. The electrocardiogram (ECG) is used to compute heart rate variability (HRV) and heart rate (HR), which indicate the contributions of sympathetic and parasympathetic activity to the heart (Appelhans and Luecken, 2008; Aslaksen and Flaten, 2008). The ECG will be continuously recorded during the stimulations from one electrode attached to the upper part of the left chest and two electrodes attached to the lower ribs on both sides. The signals will be sampled at 1000 Hz with the BIOPAC Acqknowledge 3.7.1 software (BIOPAC Systems Inc., USA).

### Pain induction system

Thermal pain will be induced by a 30 × 30 mm thermode (metal plate) controlled by a Pathway ATS (Medoc) (TSA II, Medoc, Ramat Yishai, Israel).

### NB scenarios

**Figure 1:**
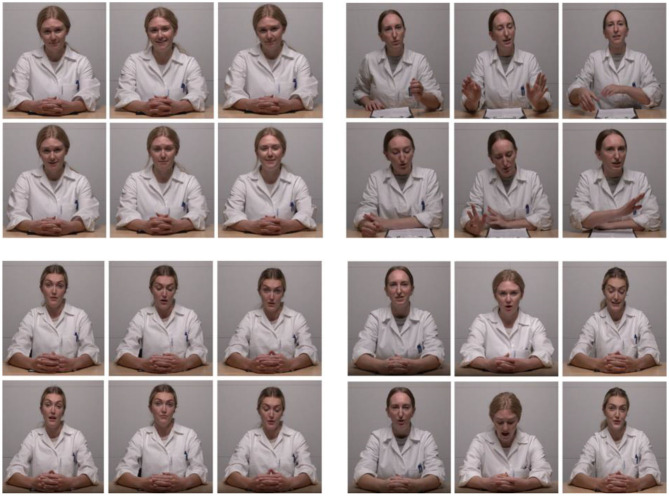
NB conditions. **Top left**: positive facial expressions (+FE); **top right**: positive body gestures (+BM); **bottom left**: neutral condition (NC); **bottom right**: positive tone of voice (+TV). Furthermore, as depicted in the **bottom right** panel, all conditions were acted by all three actors. With permission from actors.

Four NB scripts (+FE, +TV, +BM, and NC) were designed, of which three (+FE, +TV, and +BM) had one positively enhanced NB and the other as neutral as possible. Positive NBs were defined as NBs that conveyed a positive feeling to the observer (Mehrabian, 1969). Neutral NBs were defined as NBs that did not convey a specific emotion to the observer and were significantly less positive than positive NBs (Blanch-Hartigan et al., 2018). In the +FE, the VE expressed frequent smiling and nodding, enhanced eye contact (longer than a total of 5 min), more expressive eyebrow, lip, and cheek muscle movements, and more nodding. In the +TV, the VE spoke with a calm, friendly, warm, and positive tone of voice. In the +BM, the VE leaned toward the camera more frequently (to imply closer proximity to the participant) and had elaborate and expressive hand movements such as indexing, counting with fingers, and indicating sizes, timelines, and shapes with hands. Other than the positively enhanced NBs, the other NBs were kept as neutral as possible. In the NC, all NBs were kept neutral; therefore, the VE showed a flat face without smiling and without enhanced eye contact, used a monotonous tone of voice, and displayed a straight body posture without moving the hands or the torso (see Figure 1).

### Videotaped experimenters

Three Norwegian female professional actors in the age range of 26–32 played VEs. The actors were typecast to fit a usual health personnel stereotype (Mercer et al., 2008), wearing a white lab coat and light makeup. All NB conditions were played by all three actors (Figure 1). Before recording, each actor received about 10 h of training with the playscripts. VEs have been used in previous studies (Hunter et al., 2014; Ruben et al., 2017). To ascertain the validity of NBs, all video conditions were played by all actors.

### Research assistants

Three female assistants will conduct the experiment. Every assistant will test 26–28 participants. Using written instruction, the assistants will be trained to conduct the experiment and how to control their verbal and non-verbal interactions. The assistants then run a simulation experiment with the first author (HD), and if successful, they will start testing the participants.

### Blinding of assistants

Two types of information about the creams will be given to the assistants. For half of the participants, the assistants will be told that that half of the creams are active pain-relieving creams and the other half placebo creams; therefore, there will be a 50% chance for each participant to receive an active pain-relieving cream; however, you (the assistant) would not know which participant receives which cream. These assistants are hereafter called uncertain information assistants (UI assistants). For the second half of participants, the assistant will be told that all creams are active pain-relieving creams; therefore, all participants will receive the active pain-relieving cream. These assistants are hereafter called certain information assistants (CI assistants). The effects of the type of information told to assistants will be tested under the following exploratory hypotheses: (5) CI assistants will elicit larger placebo effects than UI assistants. (6) Participants tested with CI assistants will have lower stress and arousal than participants tested with UI assistants.

### Conditioning of assistants

To strengthen the belief about the effectiveness of the creama, conditioning will be performed on CI assistants with an active pain-relieving cream (lidocaine 5%), identical to the placebo. To do so, the assistants will undergo a conditioning with two phases, first without the lidocaine cream and then with the lidocaine cream. The stimulation will be a thermal stimulation, starting at 32^o^C, with an increase rate of 0.25^o^C /s, ascending until the assistant reports a pain intensity of “5” on an NRS. For the second time, a 5% lidocaine cream will be administered on the assistants' forearms with a 15-min waiting time for the cream to take effect, and then, an identical ascending thermal stimulation will be induced until the assistant reports a pain intensity equal to “5.” Afterward, the results of both pain stimulations will be shown to the assistants to prove that the cream made them tolerate a higher stimulation.

### Procedure

Eligible participants will be told that the purpose of the study is to investigate the psychological and physiological reactions to thermal pain stimulation and an over-the-counter heat pain-relieving cream. The participants will be told that the experiment is conducted by VEs; however, there will be an assistant present in the room who will carry out the experiment but will have limited interaction with participants to avoid distraction. On the testing day, the participant takes a seat on a chair, where a 70-inch screen is placed in front of them at a distance of about 2 meters. The assistant is present in the room but outside the visual field of the participant. Two VEs guide the experiment. The screen displays the first VE who informs the participant about the experiment, rating scales, physiological recordings, and tasks. Afterward, the participant will go through the calibration phase. In the calibration, the participant will undergo three ascending heat stimulations. The painful stimulus will be calibrated to a pain level of “5” to allow the observation of both the reduction and elevation of pain levels, to test the effects of NBs on both reductions and elevations of pain (Adamczyk et al., 2022). The scoring of the intensity and unpleasantness of pain is described by Price et al. (1983; for the descriptions, see Supplementary material, page 9). The VE instructs participants to allow the thermode to reach a painful temperature and let it maintain that temperature. The painful stimulus will be individually calibrated to reduce inter-individual differences in pain (e.g., Fillingim, 2005). The temperature in the thermode will be equivalent to a pain level of “5” (i.e., described as moderately painful), which has been found by the method of ascending limits: The painful stimulus will start at 32^o^C, with an increase rate of 0.25^o^C/s until the participant reports pain equal to “5” on the NRS. Three ascending stimuli will be presented, and the assistant will change the place of the thermode on the arm after each stimulation. Pain equal to “5” is determined as the average stimulus intensity where the participant reports pain of “5”. This temperature level is presented in the pre- and post-tests. This procedure will be repeated two more times, and for each time, the assistant will change the position of the thermode on the participant's arm. In the pre-test, the VE guides the participant to undergo a 4-min thermal stimulation with an individually calibrated intensity of level “5”. After 30 s, 2 min, and 4 min of the pain stimulation, the VE asks the participant to report the pain intensity and unpleasantness on an NRS, from no pain at all that is anchored to “0,” to “5” anchored to moderately painful, and to “10” as the worst pain possible. Thereafter, the participant will rest for 4 min. After the pre-test, the experimental manipulation begins.

**Figure 2:**
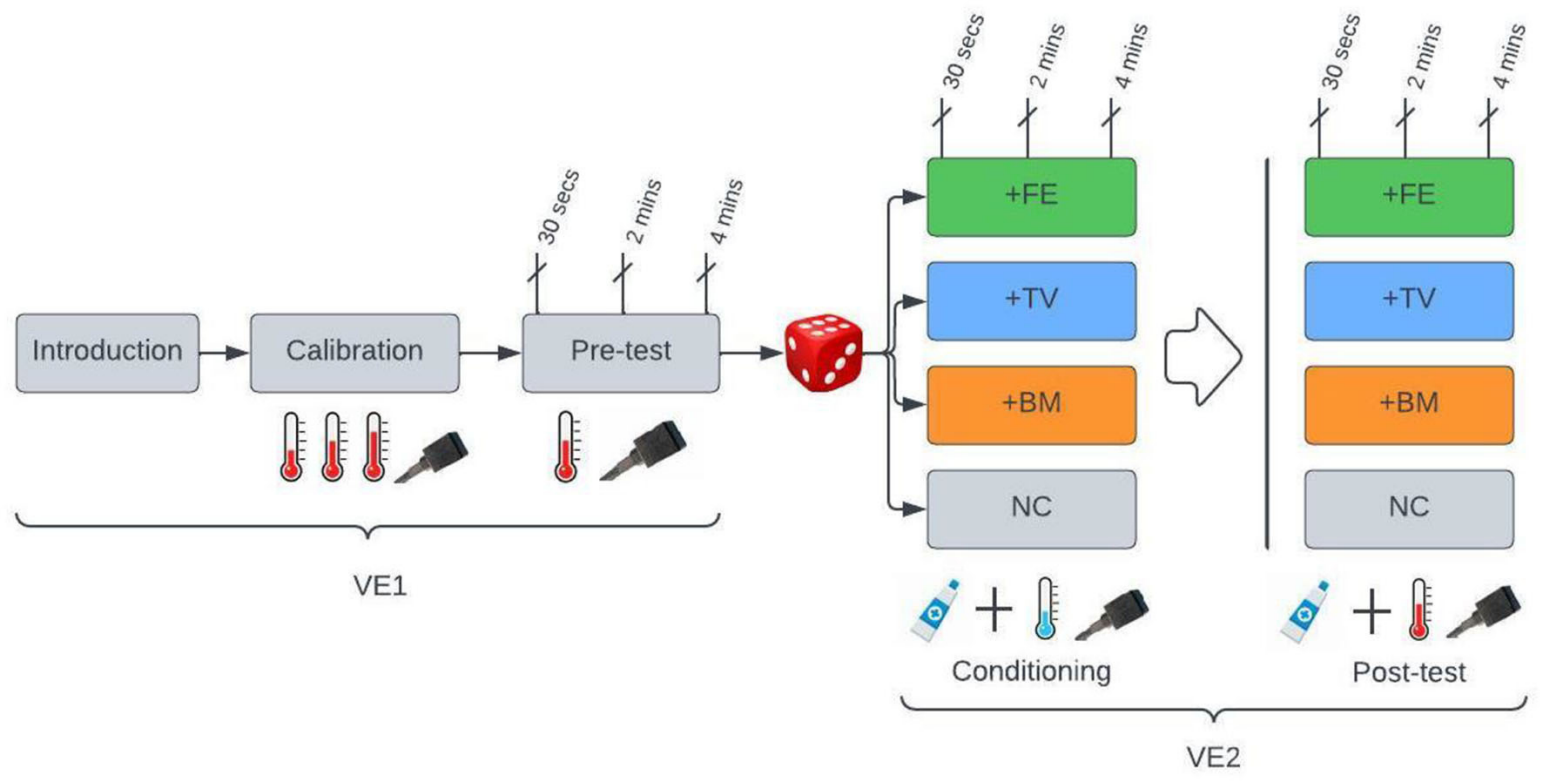
Experimental procedure. Gray boxes, neutral NBs are displayed by VEs; VE1, the first videotaped experiment that shows only neutral NBs; VE2, the second VE that has either positive or neutral NBs. +FE: positive facial expressions; +TV: positive tone of voice; +BM: positive body movements; NC: neutral condition. During the calibration, the intensity of the thermal pain (illustrated by a thermometer) will be calibrated to the individual average of pain intensity using three ascending stimulations. During the pre-test, the individually calibrated painful stimulation will be induced. Next, participants will be randomly assigned to one of the four groups. During the conditioning, a placebo cream will be applied with suggestive information from the VE2, and unbeknownst to the participant, the pain stimulation will be lowered from an intensity of “5” to “3.” In the +FE positive facial expressions, in the +TV positive tone of voice, in the +BM positive body movements, and in the NC, neutral NBs will be displayed by VE2. Each group will have two VE2s, and the participants will be randomly assigned to one of them. The post-test is identical to conditioning, with the only difference that the stimulation intensity of “5,” the same as in the pre-test, will be induced in the post-test. The pain intensity and unpleasantness will be recorded at 30 s, 2 min, and 4 min of the stimulation, and the stress and arousal will be recorded before and after the stimulations.

Prior to the conditioning phase, the second VE will be displayed to the participant and introduce the placebo cream. The verbal information about the cream is as follows: “before the next pain stimulation, you will receive a pain-relieving cream. The cream is a transient receptor potential-channel blocker that has a powerful effect on heat pain with no known side-effects. In a couple of seconds, the assistant will administer the cream on your arm, gives it 10 min to work, and then induces the stimulation. Then, you should report how much pain intensity and unpleasantness you feel.” The VE conveys this information while expressing the NBs that correspond to the group the participant is assigned to. The assistant applies the cream and mounts the thermode on the arm. Unbeknownst to the participant, the assistant lowers the temperature from the intensity of “5” to “3.” The pain level “3” will be induced for 4 min. After 30 s, 2 min, and 4 min of stimulation, the participant reports the pain intensity and unpleasantness. The experimental procedure in the post-test will be identical to the conditioning, except that the pain stimulation will be equal to “5”. Stress and arousal will be recorded before and after the stimulations (Figure 2).

### Ethics

The study is approved by regional committees for medical and healthcare research ethics of Norway (REK; project number: 71525) and the Norwegian Centre for Research Data (NSD; project number: 167011).

### Statistical power

The change score from pre-test to post-test within each condition and the difference between the positively enhanced conditions and the control condition will be analyzed. In a similar between-group study, Aslaksen et al. (2011) found an effect size of 0.478 (Natural history group mean = 3.42, *SD* = 1.52, Placebo group mean = 2.73, *SD* = 1.37) for a placebo effect in pain unpleasantness. The statistical strength is 0.5 in the present study, and the alpha level is set at 0.05. With an expected effect size of 0.478 and an estimated sample size of 80 participants (four groups; 20 participants per group), the study will have a statistical strength of approximately 0.5 (Cohen, 2013).

### Statistical analysis

Repeated measures ANOVA and linear regressions will be used to analyze the data. To simplify the analyses, the change score, i.e., the pre-test subtracted from the post-test, will be used in all analyses related to the subjective data. Mixed model analyses will be used to analyze the psychophysiological data.

### Design

We propose a mixed design with four Groups (+FE, +TV, +BM, and NC; as between factors) × 3 Timepoints (first at 30 s, second at 2 min, and third timepoint at 4 min of the stimulation), with the covariate effects of participant sex with two levels, and the knowledge of the assistants with two levels (CI and UI).

### Expected results

Three main results are expected: First, the administration of the placebo cream with the suggestive information and conditioning will result in reduction in pain and a placebo effect in all groups. Second, the amplitude of the placebo effect will be lowest in the NC compared to other groups, and lastly, the +FE will have the largest reduction in pain and placebo effect compared to other groups. Moreover, the NC will have higher stress levels (both subjective and physiological) than the other groups, and the +FE will have the lowest stress levels. Facial expressions are perhaps the most important NBs in the transmission of treatment-related expectations and beliefs from providers to the care seeker. It has been shown that the facial expressions of healthcare providers signal what expectations the doctors hold about the treatment (Valentini et al., 2014; Chen et al., 2019).

Regarding the effects of assistant expectations, it is expected that the CI assistants elicit larger placebo analgesic effects and lower stress levels compared to the UI assistants (Kaptchuk, 2001).

### Preparatory study

As a proof of concept, we tested the reliability (i.e., inter-rater reliability and internal consistency) and validity (i.e., construct validity) of the NB videos before the experiment was conducted. We tested the construct validity of NBs, i.e., that the expressed NBs are the NBs that were intended to be expressed. We asked two groups of Norwegian and US psychology students to code the NB videos based on a NB rating scale we developed. The hypotheses tested were: (a) the coding of NBs was consistent across coders (i.e., inter-rater reliability); (b) the NB manipulations were enhanced or diminished as they were intended to (i.e., construct validity of NBs); the NBs were rated similarly across actors (i.e., reliability); (c) the NB ratings were rated similarly by the Norwegian and the US coders, and (d) micro-level NBs (smile, eye contact, etc.) contributed to the macro-level ratings of dominance, positivity, and expressivity.

### NB coders

Fifteen Norwegian (11 females, 4 males; Mean age = 22.8; *SD* = 1.28) and six US (5 females, 1 male; Mean age = 21.3; *SD* = 1.54) undergraduate psychology students performed the coding. The Norwegian students performed the coding as part of their course “Bachelor Thesis in Psychology” at NTNU, in the spring semester of 2021. The US students were research assistants working in the second author's laboratory for course credit in the spring semester of 2021. The coders received training on how to use the coding log and perform the coding. The NB coding task can be done with a minimum of two coders (van Osch et al., 2017; Blanch-Hartigan et al., 2018); however, in this study, 21 coders from two different cultures were recruited.

## Measurements

### Coding log

An NB coding log was designed to rate micro-level NBs of “smiling,” “gestures,” “eye contact,” and “positivity in tone of voice”, and macro-level NB impressions of “dominance, and being in charge” “overall positivity,” and “expressivity” in each NB video. Moreover, an item regarding “attractiveness” was added to the log as a rating for physical appearance of actors. The coders were asked to rate each NB based on their general impression on a scale from “1” anchored to “not at all,” to “9,” anchored to “extremely high.” The coding log was based on the “general impression” approach (Blanch-Hartigan et al., 2018). The log eventually included “eight” items. Each of the items, except attractiveness, was operationally defined for the coders (see Supplementary material, Section Definition of NBs, for the definitions).

### Short excerpts of NB videos

As the entire length of the videos for each NB condition was about 1 h, short excerpts or thin slices of the beginning, the middle, and the end (each about 1 min) of each phase (introduction, calibration, and so forth) and the conditions (the +FE, +TV, +BM, and the NC) were extracted and attached together, making a total duration of “3” min for each phase/condition (Blanch-Hartigan et al., 2018). Since the scenarios were played by two actors, each phase had two versions, each played by one actor, therefore making a total of 14 (7 phases played × 2 actors) excerpts.

### Procedure for coding

The coding was performed in groups, and all the excerpts were rated by all coders. The coders first watched the excerpt and then rated the NB rating scale on a shared Google document. The video and the audio were played at the same time for all excerpts, but for the +TV, the audio was played without the video. The coders were told to do the coding alone and without discussion with others. Furthermore, the coders were told not to change their responses once they finished the coding. Next, the coders coded all the excerpts in one session using the coding log. The item's attractiveness was coded once for each actor.

### Statistical analyses and data screening

IBM SPSS Statistics 27.0 and STATISTICA version 7 were used. To test the reliability of the NB ratings, first, the inter-rater reliability between coders was assessed using eight intra-class coefficients (Cicchetti, 1994), and, next, internal consistency was assessed using Cronbach's alpha, separately for the Norwegian and US data, with each including eight internal consistency analyses. Next, the internal consistency and intra-class coefficients of NB ratings were tested across the three actors. The potential differences in NB ratings between actors and countries were also tested. Due to the low number of coders in the US group and the unequal *N* of the groups, two Kruskal–Wallis (K–W) non-parametric tests were separately run: the first one to test the differences in the NB ratings between countries and the second to test the differences in the NB ratings between actors.

To test the amplitude of NBs in each condition, seven one-way repeated measures ANOVA were conducted on the overall data from both the Norwegian and the US samples, as the ratings from both groups were similar. However, as there were no significant differences between the introduction, calibration, and pre-test, only the positively enhanced conditions of the +FE, +TV, +BM, and NC were tested. For these analyses, the design was with four NB conditions (+FE, +TV, +BM, and NC) as the factor on the NB ratings (i.e., dependent variables) of smile, eye contact, positivity in tone of voice, and gestures. A one-way repeated measures ANOVA was run for each NB-dependent variable.

To test the effects of micro-level NBs on impressions of dominance, overall positivity, and expressivity, repeated measures with four NBs (+FE, +TV, +BM, and NC) were used. Three one-way repeated measures ANOVAs with NBs as the factors, one for each NB impression, were performed. All significant main effects were followed up using the *Tukey* HSD.

## Results

### Descriptive

The means and standard deviations (*SDs*) of the ratings across the Norwegians and US coders are presented in Supplementary material on page 21.

### Inter-rater reliability of NB rating

Table 1 Inter-rater reliability tests using Cronbach's α and intra-class coefficients (Cicchetti, 1994) showed high internal consistency in NB coding between coders both in the Norwegian and the US groups.

**Table 1:** Cronbach's α and intra-class coefficients between the coders across coding groups and items.

**Coding items**	**Norwegian α**	**The US α**	**Norwegian ICC**	**The US ICC**
Gesture	0.99	0.98	0.99	0.98
Smile	0.99	0.92	0.99	0.96
Eye contact	0.99	0.86	0.99	0.92
Positivity in tone of voice	0.97	0.86	0.96	0.86
Dominance	0.83	0.85	0.82	0.66
Overall positivity	0.98	0.91	0.97	0.85
Expressiveness	0.98	0.93	0.96	0.91

ICC, intra-class coefficients using two-way mixed effects model. Cronbach's α: values ≥ 0.70 = acceptable reliability. ICC: values ≥ 0.60 = acceptable reliability. N of Norwegian coders: 15; N of the US coders: 6.

### Inter-rater reliability between actors

The inter-rater reliabilities of the coders for both the Norwegian and the US coders on NBs acted by actors were ≥ 0.72 for both Cronbach's alpha and ICC values.

### Differences in NB ratings between countries

The US sample had significantly higher ratings of smile [Norwegian mean rank (*MR*) = 8.67, US *MR* = 16.83, *H*(2) = 7.49, *P* = 0.006], tone of voice [Norwegian *MR* = 8.83, US *MR* = 16.42, *H*(2) = 67.41, *P* = 0.01], dominance [Norwegian *MR* = 8.90, US *MR* = 16.25, *H*(2) = 6.04, *P* = 0.01], positivity [Norwegian *MR* = 8.80, US *MR* = 16.50, *H*(2) = 6.61, *P* = 0.01], and expressivity [Norwegian *MR* = 8.67, US *MR* = 16.83, *H*(2) = 7.43, *P* = 0.006].

### Differences in NB ratings between actors

The second K–W test showed that the actors were rated similarly in gestures, eye contact, smile, tone of voice, dominance, expressivity, and overall positivity (*P* > 0.058); however, actor 1 had more gestures [*H*(2) = 10.92, *P* = 0.004] and eye contact [*H*(2) = 7.28, *P* = 0.02] than the other two; and actor 3 smiled more [*H*(2) = 16.34, *P* = 0.001]. Actors were rated differently regarding the attractiveness [*H*(2) = 19.40, *P* = 0.001].

### Construct validity of the manipulated NB conditions

There were no differences in the rating of NBs between the introduction, calibration, and pre-test (see Supplementary Table on page 21). Therefore, these phases are not analyzed further.

**Figure 3:**
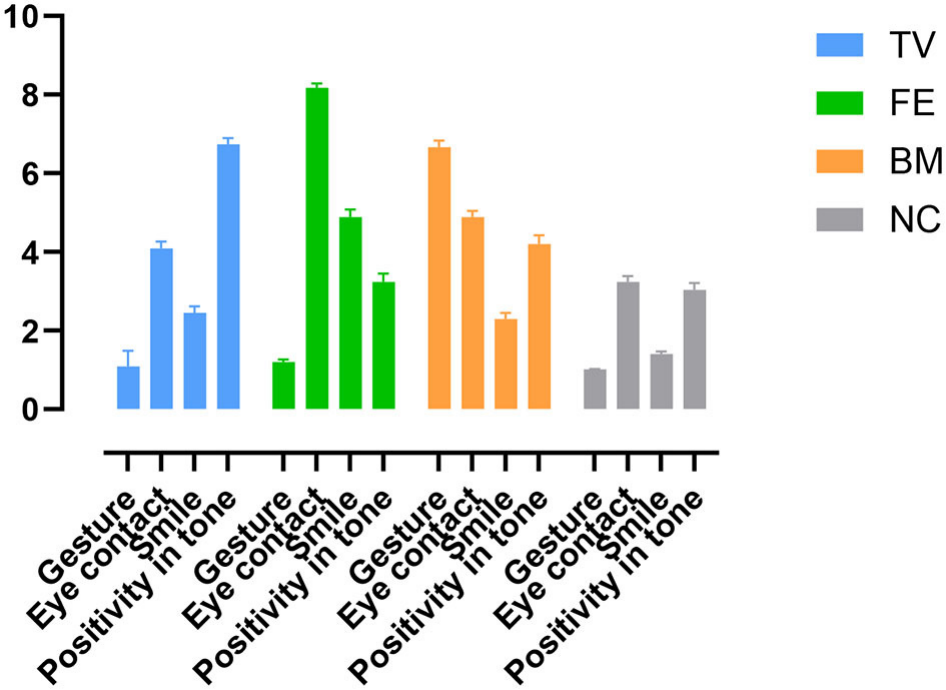
Amplitude of NBs in each NB condition. TV: positive tone of voice condition; FE: positive facial expression; BM: positive body movements; NC: neutral condition.

As expected, the +BM had higher gestures than the other conditions [*F*_(3, 18)_ = 225.74, η^2^ = 0.97]. The +FE had higher eye contact [*F*_(3, 18)_ = 97.29, η^2^ = 0.94] and smiles [*F*_(3, 18)_ = 127.96, η^2^ = 0.95] than the other conditions. The +TV had higher positivity in tone of voice than the other conditions [*F*_(3, 18)_ = 64.56, η^2^ = 0.91]. The +BM and +TV had higher smiles than the NC (*P*s < 0.001), and the +FE and +BM had higher positivity in tone of voice than the NC (*P*s < 0.001). The +FE was more positive in tone of voice compared to the +BM (*P* = 0.003), and the +BM had higher eye contact compared to the +TV (*P* = 0.049) (Figure 3).

### Effects of micro-level NBs on dominance, expressivity, and positivity

#### Dominance

The significant main effect of Condition [*F*_(2.02, 40.56)_ = 7.57, η^2^ = 0.27] was due to higher dominance in the +FE (*P* = 0.003) and +BM (*P* = 0.007) compared to the NC. There was also higher dominance in the +FE (*P* = 0.009) and +BM (*P* = 0.020) compared to the +TV. No other comparisons were significant.

#### Overall positivity

The significant main effect of Condition [*F*_(2.55, 51.09)_ = 53.26, η^2^ = 0.72] was due to higher overall positivity in the +TV (*P* = 0.0002), +FE (*P* = 0.0002), and +BM (*P* = 0.0002) compared to the NC. There was also higher overall positivity in the +FE compared to the +TV (*P* = 0.041) and the +BM (*P* = 0.0002). No other comparisons were significant.

#### Expressivity

**Figure 4:**
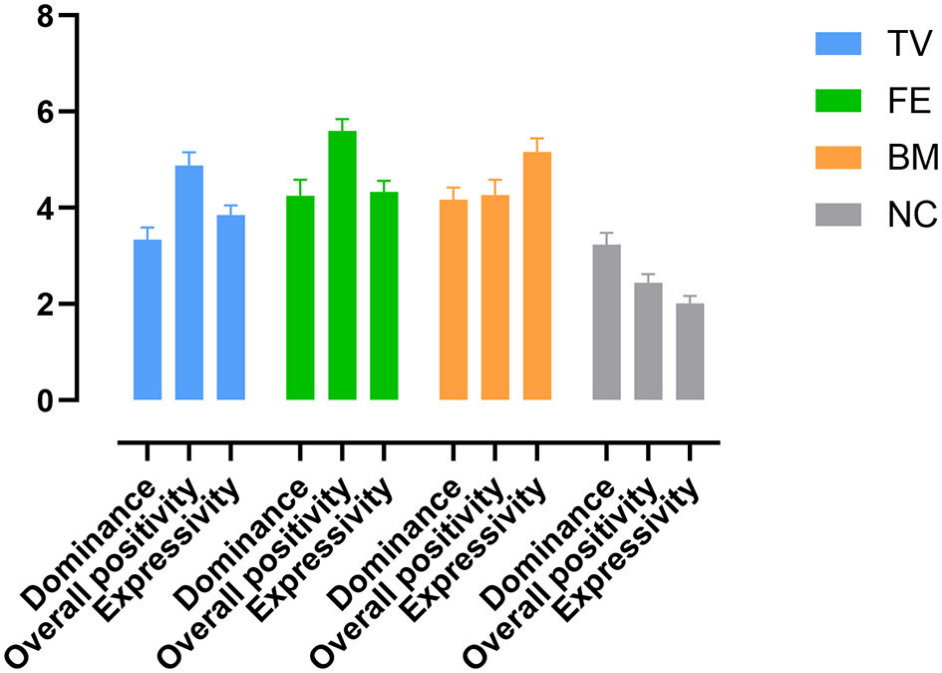
Amplitude of dominance, positivity, and expressivity in NB conditions. TV: positive tone of voice; FE: positive facial expression; BM: positive body movements; NC: neutral condition.

The significant main effect of Condition [*F*_(2.91, 58.34)_ = 58.06, η^2^ = 0.74] was due to higher expressivity in the +TV (*P* = 0.0002), +FE (*P* = 0.0002), and +BM (*P* = 0.0002) compared to the NC. There was also higher expressivity in the +BM compared to the +TV (*P* = 0.0002) and the +FE (*P* = 0.008). No other comparisons were significant (Figure 4).

## Discussion

The main findings were that first, both the NB video conditions and the NB coding log held acceptable reliability and construct validity, and second, all three positively enhanced NB conditions increased ratings of overall positivity and expressivity compared to the neutral condition. A positive tone of voice did not increase ratings of dominance compared to neutral NBs, but positive facial expressions and positive body movements did. Positive facial expressions increased ratings of overall positivity more than the other positive NBs. Positive body movements, on the other hand, increased ratings of expressivity more than the other NBs.

The moderate-to-high internal consistency and intra-class coefficients between the coders on the ratings of NBs suggested that the scale was successful at screening both the micro- and macro-level NBs, implying that simple NB rating scales that rely on general impressions can reliably capture the amplitude of NBs at both micro- and macro-levels (Blanch-Hartigan et al., 2018).

Regarding the ICC values, coders had acceptable levels of reliability; however, reliability was lower for ratings of dominance (≥ 0.66). The coders had medium-to-high consensus in ratings of NBs between actors. The overall results support the similarity in NBs between the actors, although individual differences in the expression of NBs existed, mostly in the amplitude and extent of the NBs, and therefore, not likely to damage the consistency. Moreover, this level of variability may be a characteristic of non-verbal communication, as an NB, for instance a smile, cannot be identical between two persons. Therefore, we believe that for ecological validity, this type of difference is acceptable and important for generalizability.

Even though the US group rated the NBs more positively than the Norwegian group did, both coding groups rated the micro- and macro-level NBs in the same direction. This is in line with research showing that the similarity in perception and judging of NBs is pancultural (e.g., that a smile is usually a positive NB in most cultures) (Ambady et al., 2000; Elfenbein and Ambady, 2002; Matsumoto, 2006a). However, the US coders had higher ratings than the Norwegian coders did, which is in line with documents suggesting that the judgment about the intensity of expressed NBs might vary across cultures (Ekman et al., 1987).

There were differences in the rating of physical attractiveness between actors, and physical attractiveness can affect social interactions (Reis et al., 1980, 1982) and placebo effects (e.g., Yan et al., 2018). The main aim of this experiment is to test the effects of the NBs of caregivers on the amplitude of pain reports and placebo effects. Therefore, to control for the effects of attractiveness, two actors with the higher and lower ratings of physical attractiveness will be used in each NB group of the +FE, +TV, +BM, and the NC. Thus, participants in each group will either see the actor with a higher rating or lower rating of attractiveness, and the sum of the two sub-groups will be used in the analyses. This approach may control for the confounding effects of attractiveness.

The NBs were expressed as intended. That is, the +FE had more smiling and eye contact, the +TV had higher positivity in tone of voice, and the +BM had more positive body movements and gestures compared to the NC and other conditions. The introduction, calibration, and pre-test were like the NC, having the lowest rates of NBs. This indicates that the NBs were as they were intended to be. So, in neutral conditions (introduction, calibration, pre-test, and the NC), the NBs were low as intended, and in positive conditions (+FE, +TV, and +BM), the NB(s) that were at the focus had the highest level, confirming the validity of the NB conditions. However, in positive NBs, NBs other than the NB of the focus increased as well. For example, in +BM, which had the highest gestures, eye contact was also increased. This can be due to the quality of the NBs, as some NBs can affect the other NBs (Dittmann, 1972); for example, tone of voice is affected when a welcoming body posture or a leaning forward is expressed (Dittmann and Llewellyn, 1969). Similarly, an increase in smiling was observed in the +TV, which can be partly explained by the physiological structure of the muscles involved in the production of certain NBs, such as the tone of voice and its effects on facial expressions (e.g., Campanella and Belin, 2007). However, the NBs of the focus were still the highest among the other NBs, confirming the validity of the NB manipulations.

Moreover, the increase in other NBs along with the NB of the focus suggests that the harmony between the NBs was not damaged, as even though the NB of the focus was enhanced more than the other NBs, the other NBs were accordingly increased too. Therefore, the manipulations are not likely to have produced incongruency between NBs. It has been shown that the incongruency between the communication channels (verbal and non-verbal) hinders the transmission of the message and emotions (Gorawara-Bhat et al., 2017), but the incongruency between the NBs has not been investigated very much. In a separate study, we tested whether the enhancement of NBs in current videos produced incongruency between the NBs, and the preliminary results showed no incongruency between NBs in present NBs (see Fjørstad, 2022; Nygård, 2022; Rishovd, 2022; bachelor theses).

### Effects of NBs on the impressions of dominance, overall positivity, and expressivity

Positive facial expressions, tone of voice, and body movements contributed to the impressions of overall positivity and expressivity, and positive facial expressions and body movements contributed to the impressions of dominance, whereas positive tone of voice did not, as compared to the NC.

This means that overall positivity and expressivity may be transmitted by the NBs of facial expressions, body movements, and tone of voice. Therefore, a longer gaze, more smiling, more positive and expressive body movements and gestures, and a more positive tone of voice will make the individual appear more positive and expressive. In most of previous studies, static photographs of caregiver's facial expressions are used to test the perceived impressions (Kraft-Todd et al., 2017; Necka et al., 2021); however, in this study, dynamic NBs of caregivers in three dimensions of facial expressions, body movements, and tone of voice are tested, with the results showing that higher display of such NBs, regardless of the type, contributes to the impression of overall positivity.

Facial expressions received the highest ratings of overall positivity, which is in line with previous research (e.g., Tickle-Degnen and Rosenthal, 1990; Ambady et al., 2002a) showing the role of facial expressions in the transmission of emotions. However, other positive NBs also increased the overall positivity, contradicting studies suggesting facial expressions are the only NBs to transmit positivity (e.g., Zuckerman, 1986). Our results suggest that a warmer tone of voice and open and expressive body movements contribute to perceived positivity as well as more positive facial expressions. Harrigan and Rosenthal (1983) showed that subtle changes in the body postures of clinicians, for example in their trunk angle and arm position, changed the perceived ratings of warmth, as clinicians who leaned forward more, nodded more, and had open arm positions were perceived more positively.

All positively enhanced NBs contributed to the impression of expressivity. This means that more positive facial expressions, a warmer tone of voice, and more body movements were perceived as more expressive. Moreover, positive body movements received the highest ratings of expressivity compared to all other conditions. Ekman and Friesen (1967) showed that body movements and gestures facilitated the transmission of the intensity and amplitude of that emotion, and our results showed that the body movements made the caregiver be perceived as more expressive. Therefore, our results suggest that first, adding more NBs, regardless of the type, can add to the expressivity, and second, positive body movements can be the NB that increases the expressivity the most.

Facial expressions and body movements contributed to the rating of dominance, but a positive tone of voice did not. The +FE and the +BM were both rated as positive; therefore, the perceived dominance in the +FE and +BM was not a negative impression. Dominance and being in charge can be related to impressions of competence, and previous studies have shown that positivity and competence are inversely correlated (Cuddy et al., 2004; Fiske et al., 2007) in certain contexts. However, more recently, Kraft-Todd et al. (2017) showed that perceived competence, warmth, and empathy can be positively associated in health settings. Characteristics such as higher status (Campbell et al., 2006), competence (Howe et al., 2017), and professionalism (Williams et al., 2007; Daniali and Flaten, 2019), which are usually associated with impressions of dominance, have been shown to be associated with positive treatment outcomes and lower symptom reporting. Necka et al. (2021) in an online multi-study showed that participants chose photographs of caregivers that looked more competent, and the caregivers' rated competence predicted participants' expectations about hypothetical post-procedural pain. Kraft-Todd et al. (2017) showed that participants had higher ratings of warmth for photographs of caregivers who were perceived as more competent. However, in that study, competence was tested with photographs of caregivers in white lab coats. In the present study, we tested dominance and being in charge by means of NBs and showed that more positive facial expressions and body movements add to the amplitude of perceived dominance.

A positive tone of voice did not contribute to the perceived dominance, but it did contribute to the perceived overall positivity. Previously, Ambady et al. (2002b) showed that participants could correctly tell if their caregiver had been sued by listening to content-filter audio tapes of the caregivers. It was observed that dominance in tone of voice was correlated with filing lawsuits, whereas warmth in tone of voice was not. In line with previous studies, our results suggest that a warm tone of voice does not increase perceived dominance (Riess and Kraft-Todd, 2014).

Lastly, positivity, expressivity, and dominance are three important impressions of caregivers that can have substantial influences on treatment outcomes. Our results inform about the underlying NB structures that contribute to impressions of positivity, expressivity, and dominance, with implications for patient experience and treatment outcomes. A more detailed understanding of the role of micro-level NBs in the generation of positivity, dominance, expressivity, and perhaps other medically important impressions can shed light on how placebo effects emerge.

## General discussion

This article proposed a method to systematically investigate the NBs of the caregivers in the context of pain and placebo effects, and through a preparation study, the reliability and validity of the NB manipulations were investigated. The results showed that the manipulation of the NBs by the experimenters was valid and reliable. Moreover, the contribution of micro-level NBs in the formation of impressions of dominance, positivity, and expressivity—three medically important NB impressions—was investigated.

The steps taken to manipulate the NBs were by scripting the desired NBs, training the caregivers, and testing and coding the validity of the performances. By following these steps, we elicited the desired NBs. To investigate the role of micro-NBs, it was necessary to reduce the non-verbal communication into its individual parameters. Moreover, the scripted verbal and non-verbal scenarios provided a high level of control over the communication channels of the caregivers, however, this design inevitably reduces the validity of the NBs and the generalizability of the protocol to other settings. On the other hand, this protocol shows that the NBs of the caregivers can be investigated through more rigorous and controlled paradigms. The contribution of micro-level NBs to impressions of dominance, overall positivity, and expressivity speaks to the importance of a better understanding of how impressions are formed and how these impressions affect the patients' expectations (e.g., Necka et al., 2021). Therefore, a more detailed understanding of the role of micro-level NBs in the formation of impressions is warranted (He et al., 2018).

In this experiment, all groups will receive a placebo treatment and verbal suggestions about the treatment. As there is no control group with no treatment in this design, we may not be able to exclude the reduction in pain to a placebo effect (Flaten et al., 2013). However, this design was chosen because the aim of the study was to investigate the effects of caregivers' NBs on pain reports and placebo effects. To test the effects of a factor on placebo effects, previous studies have used similar designs with no natural history control group (Flaten et al., 2006; Dutt-Gupta et al., 2007; Varelmann et al., 2010). As all groups will receive treatment plus verbal suggestions, a reduction in pain is expected in all groups. The NBs of the videotaped experimenters are the only factor that is different between the groups; thus, any difference in the reduced pain between the groups can be attributed to the non-verbal manipulations.

To strengthen the placebo effects, a conditioning procedure was also added. It has been shown that verbal suggestion about a treatment plus conditioning can enhance the generation of placebo effects (e.g., Colloca et al., 2008; Flaten et al., 2013).

## Expected limitations

Several challenges exist in conducting this experiment. First is the use of VEs, which hinder the transmission of emotions to participants. Moreover, using a videotaped caregiver will be less ecologically valid than using a real-life person. However, the use of VEs provided the highest level of control over the NB of the caregivers, and as a trade-off, this decreased the transmission of positive expectations and emotions to participants, even though previous studies reported a successful use of videotape caregivers in their experiments (e.g., Ruben et al., 2017; van Osch et al., 2017). Second, even though the assistants in the room will receive training to control their verbal and non-verbal interactions with the participants, there is still a chance that their presence affects the results (e.g., Gracely et al., 1985; Kaptchuk, 2001). However, the assistants will be given two types of information about the cream, and the effects of the assistants' information will be analyzed. Third, the expression of an emotion cannot be found in just one single NB, and oftentimes emotions are expressed through a combination of NBs (Matsumoto, 2006a). However, we did not separate the NBs but enhanced the amplitude of one or two NBs more than the others. Fourth, we proposed comparing the positively enhanced NBs with a condition with neutral NBs. Even though our preparatory study supported the validity of both the positively enhanced and the neutral conditions, the neutral conditions may still elicit emotions, as there is no true neutral NB. To partly control for the negativity effects, each participant will have two VEs, one playing the phases with neutral NBs (phases before conditioning), and the second VE playing the positively enhanced conditions (i.e., conditioning and the post-test). Fifth, along with the NB that was intentionally enhanced, some other NBs were enhanced; however, the NB of the focus was still with the highest amplitude. Furthermore, the analyses showed that the actors differed in physical attractiveness, and this could confound the effects of NBs. As mentioned before, to control for the effects of attractiveness, two actors with the higher and lower ratings of physical attractiveness will be used in each NB group. This approach may control for the confounding effects of attractiveness in the NBs. We only tested the impressions of positivity, dominance and being in charge, and expressivity. However, the combination of the manipulated NBs might have produced other impressions. Lastly, having only female actors limits the generalizability of our findings to providers of other genders.

## Ethics statement

The studies involving human participants were reviewed and approved by the Regional Committees for Medical and Healthcare Research Ethics of Norway (REK; project number: 71525) and the Norwegian Centre for research data (NSD; project number: 167011). Written informed consent was not required for the preparatory study in accordance with the national legislation and the institutional requirements. Written informed consent to participate in the pain experiment will be provided by the participants. Written informed consent was obtained from the individual(s) for the publication of any potentially identifiable images.

## Author contributions

HD and MF designed the study, drafted the manuscript, and analyzed the data. HD and MR coordinated the data collection and drafted the NB scripts, trained the actors, and the coders. HD, MF, and MR contributed to the preparation of the final manuscript. All authors contributed to the article and approved the submitted version.
