# Supplementary material for: Systematic manipulation of experimenters' non-verbal behaviors for the investigation of pain reports and placebo effects
Source: Front Psychol. 2023 Oct 31;14:1248127. doi: 10.3389/fpsyg.2023.1248127 (PMC10644817; doi:10.3389/fpsyg.2023.1248127)
Supplement: Supplementary file 1 [file Data_Sheet_1.docx]

**Supplementary materials**

# **Verbal and Nonverbal Scripts**

**Interaction with** **the experiment assistant**

***Description***: The participant will come to door to the Lab corridor. The assistant meets the participant, receives the signed consent form, and guides him/her to the test room. The participant takes a seat on the test chair, which is in front of a screen. The following is the verbal and nonverbal information the experiment assistant (EA) conveys to the participant:

| **Verbal communication of EA** | | **Nonverbal behaviors of EA** | |
| --- | --- | --- | --- |
| 1 | Hello, Welcome. | F.E^[[1]](#footnote-1)^ | Head toward the participant, one straight look at the participant for less than one second, an emotionless face |
|  |  | T.V^[[2]](#footnote-2)^ | Monotonous, not energetic, not enthusiastic |
|  |  | B.P^[[3]](#footnote-3)^ | Keeps the distance (one meter) with the participant and does not shake hands; hands are relaxed in front of the body, straight body posture |
| 2 | Are you here to participate in the study investigating the effects of ‘’heat pain on psychological and physiological responses’’? | F.E | Looking at own hands, not smiling, then one straight look for one second at the middle of the sentence, an emotionless face |
|  |  | T.V | Monotonous, not energetic, not enthusiastic |
|  |  | B.P | Keeps the distance (one meter) with the participant, hands are clenched in front of the assistant, straight body posture |
| 3 | Please follow me | F.E | No facial expressions |
|  |  | T.V | Monotonous, not energetic |
|  |  | B.P | walks ahead of the participant with the same distance, hands are relaxed aside, straight body posture |
| 4 | Please have a seat in this chair. Here is the consent form which includes detailed information about the study. This is the same form you were sent by email. If you have not already read it, please read it carefully. If you agree to participate in the study sign the last page (points where to sign) and please have a seat in this chair and give me your signed consent form.  Would you like something to drink? | F.E | Head toward the participant, a straight look at the participant for less than one second |
|  |  | T.V | Monotonous, not energetic, not enthusiastic |
|  |  | B.P | Keeps the distance (one meter) with the participant; Points at the chair with hand, straight body posture |
| 5 | As you read on the consent form, it is important that for us to know that you haven’t had coffee at least two hours before the experiment. Also please let us know if you have taken any medicine today. | F.E | Head toward the participant, a straight look at the participant for less two seconds |
|  |  | T.V | Monotonous, not energetic, not enthusiastic |
|  |  | B.P | Keeps the distance (one meter) with the participant; Points at the chair with hand, straight body posture |
| 6 | In a few moments, you will see a videotape in which two experimenters will explain everything related to the experiment today, so please watch the videos carefully.  Also, as the experiment would take about two hours, you can use the rest room now. I can show you where the rest room is. | F.E | Head toward the participant, mostly looking around, at the VR headset or other equipment, two straight looks at the participant (each one second), in the middle and at the end of the dialogue. Then looks at owns hands and avoids more eye contact. Not smiling, an emotionless face |
|  |  | T.V | Monotonous, not energetic, not enthusiastic |
|  |  | B.P | Keeps the distance (one meter) with the participant; hands are clenched in front of the body, straight body posture |
| 7 | Are you ready to watch the video? | F.E | No facial Expression, looking at the VR headset |
|  |  | T.V | Monotonous, not energetic, not enthusiastic |
|  |  | B.P | Keeps the distance (one meter) with the participant; taking the VR headset in the hand, straight body posture |

**Videotape experimenter (VE)**

1. **Introduction phase**

**Description:** the first videotaped experimenter (VE) welcomes the participant and informs about the experiment, the physiological measurements, the subjective scales, and the pain induction system. The VE will have neutral nonverbal behaviors throughout the phase, and will notify that the EA will be present but will keep the interaction with the participants at minimum.

| **Verbal information of the VE** | | **Nonverbal behavior of the VE** | |
| --- | --- | --- | --- |
| 1 | Hello,  and thank you for your participation.  As you know, this study investigates the effects of ‘’heat pain on psychological and physiological responses’’. | F.E | Head toward the camera, mostly not looking at the camera and looking around, but a straight look at the camera for one second. |
|  |  | T.V | Monotonous, not energetic, not enthusiastic. |
|  |  | B.P | Keeps the distance (one meter) with the camera; hands are clenched in front of the body; straight body posture. |
| 2 | Before we go through the details, I should inform you that the assistant present in the room will assist you throughout the experiment, however, she will not interact with you that much. | F.E | Looking at own hands, an emotionless face, sometimes looking around. Not smiling. A very quick (one second or less) look at the camera at the end. |
|  |  | T.V | Monotonous, not energetic, not enthusiastic. |
|  |  | B.P | Keeps the distance (one meter) with the camera; hands are clenched in front of the body or on the table, straight body posture. |
| 3 | This study includes several steps and in order to avoid overwhelming you, I will give you information about each task just before the task is started. During the videos, I will give you thorough information about the procedures and guide you through the experiment. The instructions are straightforward. | F.E | Head toward the camera, mostly not looking at the camera and looking around, but two straight looks (each less than one second) at the camera in the middle and at the end of the dialogue. Not smiling. |
|  |  | T.V | Monotonous, not energetic, not enthusiastic. |
|  |  | B.P | Keeps the distance (one meter) with the camera; hands are clenched in front of the assistant, straight body posture. |
| 4 | If you have any questions that could be answered by the video, the video will be played back again. However, If the videos are watched carefully, you will have enough information to perform the tasks and will be less likely to have questions. | F.E | Head toward the camera, mostly not looking at the camera and looking around, but two straight looks (each less than one second) at the camera in the middle and at the end of the dialogue. Not smiling. |
|  |  | T.V | Monotonous, not energetic, not enthusiastic. |
|  |  | B.P | Keeps the distance (one meter) with the camera; clenched hands, straight body posture. |
| 5 | Let’s start with the videos you are watching. This study is carried out using videoclips to control participants’ distraction and undesired variables. The instructions are simple, but if you needed to watch a clip again, just ask the assistant to replay the clip for you. | F.E | Head toward the camera, mostly not looking at the camera and looking around, but three straight looks (each less than one second) at the camera at the beginning, in the middle and at the end of the dialogue. Not smiling. |
|  |  | T.V | Monotonous, not energetic, not enthusiastic. |
|  |  | B.P | Keeps the distance (one meter) with the camera; hands are clenched in front of the body, straight body posture. |
| 6 | OK, now it’s time to introduce the scales that you should fill in before, during and after the heat stimulation. | F.E | No facial Expression, looking at the desk and papers, no smiles. |
|  |  | T.V | Monotonous, not energetic, not enthusiastic. |
|  |  | B.P | Keeps the distance (one meter) with the camera; hands are clenched in front of the body, straight body posture. |
| 7 | First, you should fill in four scales that ask about your stress and alertness levels. You will fill them in several times today. Each scale will show you two opposing alertness or stress adjectives that are placed at left and right ends of an 11-digit numeric rating scale. For example, the first scale is asking about your nervousness level. To answer it, you should think of how nervous you currently are and then circle the corresponding number that best describes your nervousness level on the numeric rating scale starting from ‘’0’’ which is ‘’completely calm’’, and ‘’10’’ which is ‘’maximally nervous’’. Now the assistant hands you the scale and you can start answering it. | F.E | Head toward the camera, mostly not looking at the camera and looking around, but three straight looks (each less than one second) at the camera at the beginning, in the middle and at the end of the dialogue. Not smiling. |
|  |  | T.V | Monotonous, not energetic, not enthusiastic. |
|  |  | B.P | Keeps the distance (one meter) with the camera; hands are clenched in front of the body, straight body posture. |
| 8 | The second scale is asking about your tenseness level. To answer it, you should think of how tense you are right now and then, on the numeric rating scale, circle the corresponding number that best describes your tenseness level. Here, ‘’0’’ is ‘’completely relaxed’’, and ‘’10’’ is ‘’maximally tensed’’. Let’s answer the scale. | F.E | Head toward the camera, mostly not looking at the camera and looking around, but three straight looks (each less than one second) at the camera at the beginning, in the middle and at the end of the dialogue. Not smiling. |
|  |  | T.V | Monotonous, not energetic, not enthusiastic. |
|  |  | B.P | Keeps the distance (one meter) with the camera; hands are clenched in front of the body, straight body posture. |
| 9 | The third scale is asking about your sleepiness level. To answer it, you should think about how sleepy you are right now and then, on the numeric rating scale, circle the corresponding number that best describes your sleepiness level. Here, ‘’0’’ is ‘’completely sleepy’’, and ‘’10’’ is ‘’maximally awake’’. Let’s answer the scale. | F.E | Head toward the camera, mostly not looking at the camera and looking around, but three straight looks (each less than one second) at the camera at the beginning, in the middle and at the end of the dialogue. Not smiling. |
|  |  | T.V | Monotonous, not energetic, not enthusiastic. |
|  |  | B.P | Keeps the distance (one meter) with the camera; hands are clenched in front of the body, straight body posture. |
| 10 | The fourth scale is asking about your energy level. To answer it, you should think of how energetic you are right now and then, on the numeric rating scale, circle the corresponding number that best describes your energy level. Here, ‘’0’’ is ‘’completely tired’’, and ‘’10’’ is ‘’maximally energetic’’. Let’s answer the scale. | F.E | Head toward the camera, mostly not looking at the camera and looking around, but three straight looks (each less than one second) at the camera at the beginning, in the middle and at the end of the dialogue. Not smiling. |
|  |  | T.V | Monotonous, not energetic, not enthusiastic. |
|  |  | B.P | Keeps the distance (one meter) with the camera; hands are clenched in front of the body, straight body posture. |
| 11 | Now, it’s time to answer a personality scale with 10 statements. After reading each statement, you should think about how well the statement describes your personality. You can choose an answer on a five-point Likert scale starting from ‘’Strongly disagreed’’ and ending with ‘’Strongly agreed’’. Now the assistant will hand you the questionnaire and you can start answering it.  *** | F.E | Head toward the camera, mostly not looking at the camera and looking around, but three straight looks (each less than one second) at the camera at the beginning, in the middle and at the end of the dialogue. Not smiling. |
|  |  | T.V | Monotonous, not energetic, not enthusiastic. |
|  |  | B.P | Keeps the distance (one meter) with the camera; hands are clenched in front of the body, straight body posture. |
| 12 | Another scale describes 30 different painful experiences. Read each description and think about how scared you would be to experience the pain associated with each description. If there is a description of painful situation that you have never experienced, just answer as you would expect the fear to be if you had such an experience. You can choose an answer on a five-point Likert scale starting from ‘’Not at all’’ and ending with ‘‘Extremely painful’’. Now the assistant will hand you the questionnaire and you can start answering it.  *** | F.E | Head toward the camera, mostly not looking at the camera and looking around, but three straight looks (each less than one second) at the camera at the beginning, in the middle and at the end of the dialogue. Not smiling. |
|  |  | T.V | Monotonous, not energetic, not enthusiastic. |
|  |  | B.P | Keeps the distance (one meter) with the camera; hands are clenched in front of the body, straight body posture. |
| 13 | OK, let’s have a look at the Medoc system that produces safe heat stimulation. The Medoc transfers the heat stimulation to a metal plate thermode. The thermode will be mounted on your right arm. I will provide more information before using it. | F.E | Head toward the Medoc machine, mostly not looking at the camera and looking around, but two straight looks (each less than one second) in the middle and at the end of the dialogue. Not smiling. |
|  |  | T.V | Monotonous, not energetic, not enthusiastic. |
|  |  | B.P | Keeps the distance (one meter) with the camera; hands are clenched in front of the body, straight body posture. |
| 14 | Ok, here are the disposable electrodes, which are risk-free and used to take your electrophysiological recordings. After this video is stopped, the assistant will attach three electrodes on your chest and two electrodes on the fingers of your left hand. Before attaching the electrodes, the assistant will apply an electrode gel to improve your skin conductivity. Both the electrodes and the gel are safe and harmless. | F.E | Head toward the electrodes, mostly not looking at the camera and looking around, but two straight looks (less than one second) in the middle and at the end of the dialogue. Not smiling. |
|  |  | T.V | Monotonous, not energetic, not enthusiastic. |
|  |  | B.P | Keeps the distance (one meter) with the camera; hands are clenched in front of the body, straight body posture. |
| 15 | Before the electrodes can be attached, you have to change to a standard t-shirt which the assistant hands you in a minute. This is to ensure that the contact of the clothes with the electrodes does not distort the recording process. After you received the t-shirt, you can use the divider to change and the assistant waits outside until you declare ready to continue the experiment. After you changed, the assistant will come back and starts the process to attach the electrodes. Now the assistant will hand you the t-shirt and guide you to the changing divider. | F.E | Head toward the electrodes, mostly not looking at the camera and looking around, but one straight looks (less than one second) in the middle of the dialogue (when saying ‘if you wish’). Not smiling. |
|  |  | T.V | Monotonous, not energetic, not enthusiastic. |
|  |  | B.P | Keeps the distance (one meter) with the camera; hands are clenched in front of the body, straight body posture. |
| 16 | Now it is time to attach the physiological electrodes. Please keep in mind that the assistant will not interact with you that much during the process. After the videos are attached, you will see the rest of the videos. | F.E | Head toward the camera, mostly not looking at the camera and looking around, but one straight looks (less than one second) at the camera at the end of the dialogue. Not smiling. |
|  |  | T.V | Monotonous, not energetic, not enthusiastic. |
|  |  | B.P | Keeps the distance (one meter) with the camera; hands are clenched in front of the body, straight body posture. |
| 17 | Now that the electrodes are attached, you should remember to stay still in the chair, without moving your hands and body.  To fill in the scales from now on, you can verbally say your desired answers to the assistant, and she will record them. | F.E | No facial Expression, looking at the Medoc machine, no smiles. |
|  |  | T.V | Monotonous, not energetic, not enthusiastic. |
|  |  | B.P | Keeps the distance (one meter) with the camera; hands are clenched in front of the body, straight body posture. |
| 18 | Now, let’s prepare for the next step.  ( at least five minutes time to get the baseline recording) | F.E | No facial Expression, looking at the Medoc machine, no smiles. |
|  |  | T.V | Monotonous, not energetic, not enthusiastic. |
|  |  | B.P | Keeps the distance (one meter) with the camera; hands are clenched in front of the body, straight body posture. |

1. **Pain Calibration phase**

**Description:** the VE asks informs about the pain stimulation procedure, and how to report pain intensity on a Numeric Rating Scale (NRS).

| 1 | Before talking about this step, you should report the stress and alertness scales you just filled in a few minutes ago. Like before, you should rate four scales that ask about your stress and alertness levels. Read the question for each scale and then think of a number, from ‘’0’’ to ‘’10’’, that best describes your current state. Report that number on the numeric rating scale. | F.E | Head toward the camera, mostly not looking at the camera and looking around, but three straight looks (each less than one second) at the camera at the beginning, in the middle and at the end of the dialogue. Not smiling. |
| --- | --- | --- | --- |
|  |  | T.V | Monotonous, not energetic, not enthusiastic. |
|  |  | B.P | Keeps the distance (one meter) with the camera; hands are clenched in front of the body, straight body posture. |
| 2 | The stress and alertness scales will be displayed to you one at a time. For each scale, you should verbally report the number, on a scale from ‘’0’’ to ‘’10’’, that best describes your status. Let’s see the first scale. |  |  |
| 3 | Ok, now it’s time to give you information about the heat stimulation procedure. As said earlier, this Medoc machine produces heat stimulation that is transferred to your right forearm by the thermode. The assistant mounts the thermode on your arm and induces the pain stimulation. The temperature on the thermode will increase gradually until you report pain intensity equal to ‘’seven’’ on an 11-digit Numeric Rating Scale. | F.E | Head toward the camera, mostly not looking at the camera and looking around, but two straight looks (each less than one second) at the camera at the beginning, in the middle and at the end of the dialogue. Not smiling. |
|  |  | T.V | Monotonous, not energetic, not enthusiastic. |
|  |  | B.P | Keeps the distance (one meter) with the camera; hands are clenched in front of the body, straight body posture. |
| 4 | To report pain intensity, you should think of how strong the pain you are feeling is, and then on a scale from zero to ten, report the number that shows the pain intensity you feel. You can see an example of this scale in front of you. An increase from ‘’zero’’ to ‘’one’’ indicates that you can feel the thermode is heating up, but it is not yet painful. So, ‘’one’’ is your pain threshold, where the stimulus starts to feel painful. ‘’Five’’ is moderately painful, and ‘’ten’’ is the worst possible pain. As the thermode heats up, the stimulation gets more painful. You should accordingly report each time the pain intensity increases by one unit, from ‘’one’’, where you start to sense the thermode heating up, to ‘’2’’, ‘’3’’, ‘’4’’ and up to ‘’7’’. | F.E | Head toward the camera, mostly not looking at the camera and looking around, but three straight looks (each less than one second) at the camera at the beginning, in the middle and at the end of the dialogue. Not smiling. |
|  |  | T.V | Monotonous, not energetic, not enthusiastic. |
|  |  | B.P | Keeps the distance (one meter) with the camera; hands are clenched in front of the body, straight body posture. |
| 5 | You will receive three pain stimulations. And before each stimulation, the assistant will change the place of thermode on your arm. Now it’s time to undergo the first heat stimulation. Let’s try it. | F.E | Head toward the camera, mostly not looking at the camera and looking around. Not smiling. |
|  |  | T.V | Monotonous, not energetic, not enthusiastic. |
|  |  | B.P | Keeps the distance (one meter) with the camera; hands are clenched in front of the body, straight body posture. |
| 6 | Now we should repeat the procedure. You will receive the stimulation and then should report the pain intensity you feel on the numeric rating scale again. Let’s try it. | F.E | Head toward the camera, mostly not looking at the camera and looking around, but two straight looks (each less than one second) at the camera in the middle and at the end of the dialogue. Not smiling. |
|  |  | T.V | Monotonous, not energetic, not enthusiastic. |
|  |  | B.P | Keeps the distance (one meter) with the camera; hands are clenched in front of the body, straight body posture. |
| 7 | OK, now we should repeat the procedure for the last time. You will receive the stimulation again and then should report the pain intensity you feel on the numeric rating scale again. Let’s try it. | F.E | No facial Expression, looking at the Medoc machine, no smiles. |
|  |  | T.V | Monotonous, not energetic, not enthusiastic. |
|  |  | B.P | Keeps the distance (one meter) with the camera; hands are clenched in front of the body, straight body posture. |
| 8 | OK, now you have finished the first phase. Let’s go through the second phase. | F.E | No facial Expression, looking at the Medoc machine, no smiles. |
|  |  | T.V | Monotonous, not energetic, not enthusiastic. |
|  |  | B.P | Keeps the distance (one meter) with the camera; hands are clenched in front of the body, straight body posture. |

1. **Pre-test phase**

**Description**: The VE informs that the participant will undergo another pain stimulation. The VE notifies that if the participant feels the heat pain became unbearable, they could push a button and the heat pain will return to the baseline temperature. The VE will describe the difference between pain unpleasantness and pain intensity and how to rate them. After 30 seconds, two, and four minutes of the pain stimulation, the participant reports the pain intensity and unpleasantness on a NRS. Then, the VE asks the participant to report the SACL. Thereafter, participant rests for four minutes.

| 1 | Before talking about this step, you will report the same alertness and stress scales you rated a few minutes ago. Like before, these four different scales ask about your stress and alertness levels. Read the question for each scale and then verbally report the number, from ‘’0’’ to ‘’10’’, that best describes your current state. ‘’report’’ that number on the numeric rating scale. Let’s see the first scale. | F.E | Head toward the camera, mostly not looking at the camera and looking around, but three straight looks (each less than one second) at the camera at the beginning, in the middle and at the end of the dialogue. Not smiling. |
| --- | --- | --- | --- |
|  |  | T.V | Monotonous, not energetic, not enthusiastic. |
|  |  | B.P | Keeps the distance (one meter) with the camera; hands are clenched in front of the body, straight body posture. |
| 2 | Ok, now it’s time to undergo another heat pain stimulation. This time the heat stimulation will last for four minutes. For this stimulation, the temperature increases rapidly. | F.E | Head toward the camera, mostly not looking at the camera and looking around, but three straight looks (each less than one second) at the camera at the beginning, in the middle and at the end of the dialogue. Not smiling. |
|  |  | T.V | Monotonous, not energetic, not enthusiastic. |
|  |  | B.P | Keeps the distance (one meter) with the camera; hands are clenched in front of the body, straight body posture. |
| 3 | After 30 seconds, two minutes, and four minutes of the pain stimulation, you should report the pain intensity and pain unpleasantness you feel. | F.E | No facial Expression, looking at the Medoc machine, no smiles. |
|  |  | T.V | Monotonous, not energetic, not enthusiastic. |
|  |  | B.P | Keeps the distance (one meter) with the camera; hands are clenched in front of the body, straight body posture. |
| 4 | As said earlier, the pain intensity is how strong the pain feels. The pain unpleasantness is how unpleasant or disturbing the pain is for you. The distinction between these two aspects of pain is clearer if you think of listening to a sound, such as a radio. As the volume of the sound increases, I can ask you how loud it sounds or how unpleasant it is to hear it. The intensity of pain is like loudness; the unpleasantness of pain depends not only on intensity but also on other factors which may affect you.  Although some pain sensations may be equally intense and unpleasant, we would like you to judge the two aspects independently. Using the same scale from ‘’0’’ to ‘’10’’, you should first report the relative intensity of your pain sensation; and then the relative unpleasantness. | F.E | Head toward the camera, mostly not looking at the camera and looking around, but four straight looks (each less than one second) at the camera, one at the beginning, two in the middle and one at the end of the dialogue. Not smiling. |
|  |  | T.V | Monotonous, not energetic, not enthusiastic. |
|  |  | B.P | Keeps the distance (one meter) with the camera; hands are clenched in front of the body, straight body posture.  (The tech guy get control of the remote) |
| 5 | Before undergoing the heat pain stimulation, you will report the same stress and alertness scales you rated a few minutes ago. Like before, these four different scales ask about your stress and alertness levels. Read the question for each scale and then report the number, from ‘’0’’ to ‘’10’’, that best describes your current state. Report that number from the numeric rating scale *** | F.E | Head toward the camera, mostly not looking at the camera and looking around, but two straight looks (each less than one second) at the camera in the middle and at the end of the dialogue. Not smiling. |
|  |  | T.V | Monotonous, not energetic, not enthusiastic. |
|  |  | B.P | Keeps the distance (one meter) with the camera; hands are clenched in front of the body, straight body posture. |
| 6 | Now, the assistant will mount the thermode on your arm, and records your physiological data for four minutes, then the technician will induce the stimulation for another four minutes. When being asked, you should report your pain intensity and then pain unpleasantness on the scale from ‘’0’’ to ‘’10’’. | F.E | Head toward the camera, mostly not looking at the camera and looking around, but one straight looks (each less than one second) at the camera, in the middle of the dialogue. Not smiling. |
|  |  | T.V | Monotonous, not energetic, not enthusiastic. |
|  |  | B.P | Keeps the distance (one meter) with the camera; hands are clenched in front of the body, straight body posture. |
| 7 | (after 30 seconds of stimulation):  Please report your pain intensity and then your pain unpleasantness on a scale from ‘’0’’ to ‘’ten’’. | F.E | Head toward the camera, mostly not looking at the camera and looking around, but one straight looks (each less than one second) at the camera, in the middle of the dialogue. Not smiling. |
|  |  | T.V | Monotonous, not energetic, not enthusiastic. |
|  |  | B.P | Keeps the distance (one meter) with the camera; hands are clenched in front of the body, straight body posture. |
| 8 | (after two minutes of stimualtion):  Please report your pain intensity and then your pain unpleasantness on a scale from ‘’0’’ to ‘’ten’’. | F.E | Head toward the camera, mostly not looking at the camera and looking around, but one straight looks (each less than one second) at the camera, in the middle of the dialogue. Not smiling. |
|  |  | T.V | Monotonous, not energetic, not enthusiastic. |
|  |  | B.P | Keeps the distance (one meter) with the camera; hands are clenched in front of the body, straight body posture. |
| 9 | (at four minutes of stimulaion):  Please report your pain intensity and then your pain unpleasantness on a scale from ‘’0’’ to ‘’ten’’. | F.E | Head toward the camera, mostly not looking at the camera and looking around, but one straight looks (each less than one second) at the camera, in the middle of the dialogue. Not smiling. |
|  |  | T.V | Monotonous, not energetic, not enthusiastic. |
|  |  | B.P | Keeps the distance (one meter) with the camera; hands are clenched in front of the body, straight body posture. |
| 10 | Now you should report the same alertness and stress scales you rated a few minutes ago. Like before, these four different scales ask about your stress and alertness levels. Read the question for each scale and then report the number, from ‘’0’’ to ‘’10’’, that best describes your current state. Report that number from the numeric rating scale. | F.E | Head toward the camera, mostly not looking at the camera and looking around, but one straight looks (each less than one second) at the camera, in the middle of the dialogue. Not smiling. |
|  |  | T.V | Monotonous, not energetic, not enthusiastic. |
|  |  | B.P | Keeps the distance (one meter) with the camera; hands are clenched in front of the body, straight body posture. |
| 11 | Now you have finished this task. From the next phase on, the ‘’Lead experimenter’’ will give guide you through the rest of experiment. | F.E | Head toward the camera, mostly not looking at the camera and looking around, but one straight looks (each less than one second) at the camera, in the middle of the dialogue. Not smiling. |
|  |  | T.V | Monotonous, not energetic, not enthusiastic. |
|  |  | B.P | Keeps the distance (one meter) with the camera; hands are clenched in front of the body, straight body posture. |

1. **Conditioning**

**Descriptions**: the verbal information is identical across the groups. Please not that the section below is for the instruction for the group positive facial expressions.

**4a) Positive facial expressions**

| 1 | Hello, thank you for your participation in this study. From now on, I will give the information you need to complete the tasks. Let’s go through the first task. | F.E | Head toward the camera in a straight position, looking at the camera throughout the dialogue, affirmative blinking. Smiling. Eyebrow going up and down positively. Nodding in the second part of the dialogue (when saying I will give information…). |
| --- | --- | --- | --- |
|  |  | T.V | Monotonous, not energetic, not enthusiastic. |
|  |  | B.P | Keeps the distance (one meter) with the camera; hands are clenched in front of the body, straight body posture. |
| 2 | First, you will report the same alertness and stress scales you rated a few minutes ago. Like before, these four different scales ask about your stress and alertness levels. Read the question for each scale and then verbally report the number, from ‘’0’’ to ‘’10’’, that best describes your current state. Report that number on the numeric rating scale. Let’s see the first scale.  *** | F.E | Head toward the camera in a straight position, looking at the camera throughout the dialogue, sometimes showing affirmative blinking, eyebrow going positively up and down in an affirmative way. Smiling throughout the dialogue. Nodding for several times. |
|  |  | T.V | Monotonous, not energetic, not enthusiastic. |
|  |  | B.P | Keeps the distance (one meter) with the camera; hands are clenched in front of the body, straight body posture. |
| 3 | Before undergoing the next stimulation, the assistant will apply a heat-pain relieving cream on your arm. The cream is named ‘’Embla’’. ‘’Embla’’ is a transient receptor potential-channel blocker that has a powerful pain-relieving effect on heat pain with no known side-effects. Simply put, ‘’Embla’’ blocks the ‘’thermoreceptors’’ or heat-sensory receptors of your forearm for a short time, resulting in less pain from the heat. In a couple of seconds, the assistant will apply the ‘’Embla’’ on your arm, allowing it 10 minutes to work. Then, the assistant will mount the thermode and induce the stimulation for four minutes. After 30 seconds, two minutes and four minutes, you should report how much pain intensity and unpleasantness you feel. Let’s begin the procedure. | F.E | Head toward the camera in a straight position, looking at the camera throughout the dialogue, sometimes showing affirmative blinking, eyebrow going up and down in an affirmative way. Smiling throughout the dialogue. Nodding for several times. |
|  |  | T.V | Monotonous, not energetic, not enthusiastic. |
|  |  | B.P | Keeps the distance (one meter) with the camera; hands are clenched in front of the body, straight body posture. |
| 4 | Now you will report the same alertness and stress scales you rated a few minutes ago. Like before, these four different scales ask about your stress and alertness levels. Read the question for each scale and then report the number, from ‘’0’’ to ‘’10’’, that best describes your current state. Report that number on the numeric rating scale. Let’s see the first scale.  *** | F.E | Head toward the camera in a straight position, looking at the camera throughout the dialogue, sometimes showing affirmative blinking, eyebrow going positively up and down in an affirmative way. Smiling throughout the dialogue. Nodding for several times. |
|  |  | T.V | Monotonous, not energetic, not enthusiastic. |
|  |  | B.P | Keeps the distance (one meter) with the camera; hands are clenched in front of the body, straight body posture. |
| 5 | Now I would like you to rate how much you expect the ‘’Embla’’ will reduce the pain using an 11-digit Numeric Rating Scale, where ‘’0’’ is ‘’No pain reduction’’ and ‘’10’’ is ‘’100% pain reduction’’. Report the number that best describes your expected efficacy.  *** | F.E | Head toward the camera in a straight position, looking at the camera throughout the dialogue, sometimes showing affirmative blinking, eyebrow going positively up and down in an affirmative way. Smiling throughout the dialogue. Nodding for several times. |
|  |  | T.V | Monotonous, not energetic, not enthusiastic. |
|  |  | B.P | Keeps the distance (one meter) with the camera; hands are clenched in front of the body, straight body posture. |
| 6 | Now that you have rated your expected efficacy for ‘’Embla’’. Let’s undergo the heat stimulation. The assistant will first record your physiological measurement for four minutes and then the technician induces the stimulation for four minutes. | F.E | Head toward the camera in a straight position, looking at the camera throughout the dialogue, sometimes showing affirmative blinking, eyebrow going positively up and down in an affirmative way. Smiling throughout the dialogue. Nodding for several times. |
|  |  | T.V | Monotonous, not energetic, not enthusiastic. |
|  |  | B.P | Keeps the distance (one meter) with the camera; hands are clenched in front of the body, straight body posture. |
| 7 | (after 30 seconds since the stimulation):  Please report your pain intensity and then your pain unpleasantness on a scale from ‘’0’’ to ‘’ten’’. | F.E | Head toward the camera in a straight position, looking at the camera throughout the dialogue, sometimes showing affirmative blinking, eyebrow going positively up and down in an affirmative way. Smiling throughout the dialogue. Nodding for several times. |
|  |  | T.V | Monotonous, not energetic, not enthusiastic. |
|  |  | B.P | Keeps the distance (one meter) with the camera; hands are clenched in front of the body, straight body posture. |
| 8 | (after two minutes since the stimulation):  Please report your pain intensity and then your pain unpleasantness on a scale from ‘’0’’ to ‘’ten’’. | F.E | Head toward the camera in a straight position, looking at the camera throughout the dialogue, sometimes showing affirmative blinking, eyebrow going positively up and down in an affirmative way. Smiling throughout the dialogue. Nodding for several times. |
|  |  | T.V | Monotonous, not energetic, not enthusiastic. |
|  |  | B.P | Keeps the distance (one meter) with the camera; hands are clenched in front of the body, straight body posture. |
| 9 | (after four minutes since the stimulation):  Please report your pain intensity and then your pain unpleasantness on a scale from ‘’0’’ to ‘’ten’’. | F.E | Head toward the camera in a straight position, looking at the camera throughout the dialogue, sometimes showing affirmative blinking, eyebrow going positively up and down in an affirmative way. Smiling throughout the dialogue. Nodding for several times. |
|  |  | T.V | Monotonous, not energetic, not enthusiastic. |
|  |  | B.P | Keeps the distance (one meter) with the camera; hands are clenched in front of the body, straight body posture. |
| 10 | Now you will report the same alertness and stress scales you rated a few minutes ago. Like before, these four different scales ask about your stress and alertness levels. Read the question for each scale and then report the number, from ‘’0’’ to ‘’10’’, that best describes your current state. Report that number on the numeric rating scale. Let’s see the first scale.  *** | F.E | Head toward the camera in a straight position, looking at the camera throughout the dialogue, sometimes showing affirmative blinking, eyebrow going positively up and down in an affirmative way. Smiling throughout the dialogue. Nodding for several times. |
|  |  | T.V | Monotonous, not energetic, not enthusiastic. |
|  |  | B.P | Keeps the distance (one meter) with the camera; hands are clenched in front of the body, straight body posture. |
| 11 | Now you finished this task. Let’s go through the third phase. | F.E | Head toward the camera in a straight position, looking at the camera throughout the dialogue, sometimes showing affirmative blinking, eyebrow going positively up and down in an affirmative way. Smiling throughout the dialogue. Nodding for several times. |
|  |  | T.V | Monotonous, not energetic, not enthusiastic. |
|  |  | B.P | Keeps the distance (one meter) with the camera; hands are clenched in front of the body, straight body posture. |

**4b) Positive tone of voice and Positive body postures groups**

The verbal instructions and the neutral NB channels were identical across the groups, For positive tone of voice group the VE was instructed to play the chunks with a warm and friendly, strong, energetic, and expressively loud tone of voice; that sounds confident and enthusiastic. For the positive body posture and movements, the VE was instructed to lean forward frequently with less distance to the camera (half a meter); have expressive and elaborate hand movements (indexing, affirming, numerical listing with fingers, showing and simulating sizes and timelines). For the neutral control group, the VE was instructed to keep all their NBs as neutral as possible, therefore, head toward the camera, mostly not looking at the camera and looking around, not smiling, not nodding, with a monotonous, not energetic and mot enthusiastic tone of voice and with a one meter distance form the camera with less leaning towards the camera and with the hands clenched in front of the body.

1. **Post test**

The verbal and nonverbal scripts in post-test is identical to the conditioning phase for each group. Therefore, only the last step of the post-test which is different from the conditioning is presented. Please note that the section below is an example from positive facial expression group.

**last section**

| 1 | Before we finish the experiment, you should fill in a short scale asking how satisfied you are with your ‘’Lead experimenter’’. Please note that you should fill in this scale about me, as your Lead experimenter, and not the former videotaped experimenter. You can choose an answer on a five-point Likert scale starting from ‘‘poor’’ and ending with ‘’excellent’’. Now let’s start from the first statement. | F.E | Head toward the camera in a straight position, looking at the camera throughout the dialogue, sometimes showing affirmative blinking, eyebrow going positively up and down in an affirmative way. Smiling throughout the dialogue. Nodding for several times. |
| --- | --- | --- | --- |
|  |  | T.V | Monotonous, not energetic, not enthusiastic. |
|  |  | B.P | Keeps the distance (one meter) with the camera; hands are clenched in front of the body, straight body posture. |

## **Nonverbal behavior coding log**

| From "not at all" to "extremely" how much did the experimenter do or give off each of the following? | | | | | | | | | |
| --- | --- | --- | --- | --- | --- | --- | --- | --- | --- |
|  | 1  (Not at all) | 2 | 3 | 4 | 5 | 6 | 7 | 8 | 9  (extremely) |
| Gestures | 1 | 2 | 3 | 4 | 5 | 6 | 7 | 8 | 9 |
| Smile | 1 | 2 | 3 | 4 | 5 | 6 | 7 | 8 | 9 |
| Eye contact | 1 | 2 | 3 | 4 | 5 | 6 | 7 | 8 | 9 |
| Positive tone of voice | 1 | 2 | 3 | 4 | 5 | 6 | 7 | 8 | 9 |
| Dominant and in charge | 1 | 2 | 3 | 4 | 5 | 6 | 7 | 8 | 9 |
| General positivity | 1 | 2 | 3 | 4 | 5 | 6 | 7 | 8 | 9 |
| Expressive | 1 | 2 | 3 | 4 | 5 | 6 | 7 | 8 | 9 |
| Physical attractiveness | 1 | 2 | 3 | 4 | 5 | 6 | 7 | 8 | 9 |

### **Definitions of the NBs**

Each of the items of the coding log were operationally defined to the coders: for the item ‘smiling’, the coders were asked to look at the frequency, duration and expansiveness of smiles expressed by VEs, and then based on their general impression, rate how much smile was expressed from ‘1’ as ‘not at all’, to ‘9’ as ‘extremely high/a lot’. For ‘gestures’, the coders were trained to look for the frequency of a series of body movements and gestures as leaning forward and hand movements such as indexing, counting with fingers, showing sizes, shapes, timelines, and forms with hands, and then rate their impression of the gestures and postures displayed in each excerpt. For ‘eye contact’, the coders looked at the duration and frequency of the eye contact the VE held with the camera. The items gestures, eye contact and smile were coded with the sound off. For positivity in tone of voice, the coders were told to play only the audiotape (without the videotape file) and rate on the same scale, how positive and friendly they think the voices for each excerpt were. Dominance and being in charge was defined as a nonverbal characteristic that implies status of authority, power, social control and high rank (Argyle, 2013; Ekman et al., 2013; Zimmerman, 1976). Nonverbal expressivity was defined as the ability to clearly transmit feelings, thoughts and messages (Friedman & Riggio, 1981). Overall positivity was defined as how generally positive the experimenter seemed to be in terms of transmitting positive feelings, attitudes and a positive relationship (Ambady et al., 2000). No definition was provided for physical attractiveness, and the coders were asked to rate how attractive they thought the actors were. The coders were asked to do the coding for all four conditions individually, and without consulting with each other. Also, the coders were told not to change the responses once they finished the coding. Next, the coders coded all the excepts in one session using the coding log.

**Mean and SDs of the NB ratings for each phase across coder groups**

| Excerpts |  | **GE** (M; SD) | **SM** (M; SD) | **EC** (M; SD) | **TV** (M; SD) | **DO** (M; SD) | **OP** (M; SD) | **EX** (M; SD) |
| --- | --- | --- | --- | --- | --- | --- | --- | --- |
| **Intro** | ***CodingG***  *Norway* | 1.44; .16 | 1.67; .46 | 3.71; .84 | 3.35; .49 | 3.06; .24 | 2.82; .66 | 2.66; .50 |
|  | *USA* | 1.05; .09 | 2.55; .67 | 4.27; .67 | 4.00; 1.30 | 3.66; .76 | 4.16; 1.04 | 2.88; .67 |
| **Cal** | *Norway* | 1.35; .07 | 1.60; .43 | 3.00; 1.16 | 3.26; .40 | 2.82; .07 | 2.68; .53 | 1.80; .11 |
|  | *USA* | 1.05; .09 | 1.82; .20 | 3.27; 1.00 | 3.70; .75 | 3.05; .50 | 3.11; .63 | 2.72; .38 |
| **Pre-test** | *Norway* | 1.11; .38 | 1.48; .39 | 2.82; .61 | 3.42; .55 | 2.80; .23 | 2.48; .50 | 2.13; .23 |
|  | *USA* | 1.00; .0 | 2.00; .44 | 3.38; .85 | 4.27; 1.0 | 4.16; .60 | 3.83; 1.01 | 3.00; .28 |
| **+TV** | *Norway* | 1.13; .06 | 2.22; .90 | 3.91; .73 | 6.60; .00 | 3.02; .07 | 4.48; .21 | 3.57; .30 |
|  | *USA* | 1.00; .0 | 3.00; .50 | 4.66; .44 | 7.11; .78 | 4.16, .44 | 5.88; .19 | 4.50; .66 |
| **+FE** | *Norway* | 1.28; .07 | 6.17; 1.12 | 8.24; .23 | 4.84; .48 | 3.80; .24 | 5.31; .68 | 4.04; .23 |
|  | *USA* | 1.00; .0 | 7.55; .67 | 8.00; .33 | 6.66; .76 | 5.38; .41 | 6.33; .60 | 5.05; .34 |
| **+BM** | *Norway* | 6.62; .60 | 1.93; .88 | 4.71; .44 | 3.68; .44 | 3.75; .60 | 3.75; .64 | 4.75; .74 |
|  | *USA* | 6.72; .67 | 3.22; .97 | 5.27; .53 | 5.55; .67 | 5.22; .78 | 5.50; .60 | 6.38; .67 |
| **NC** | *Norway* | 1.31; .07 | 1.33; .46 | 3.13; .48 | 2.68; .40 | 2.91; .21 | 2.13; .34 | 1.75; .23 |
|  | *USA* | 1.11; .09 | 1.61; .34 | 3.66; .33 | 3.88; 1.25 | 4.05; .34 | 3.16; .88 | 2.38; .38 |

**Note**. Mean; SDs. GE: gestures; SM: smiling; EC: eye contact; TV: tone of voice (rating item); DO: dominance; OP: overall positivity; EX: expressivity; Intro: introduction; CodingG: coding group; Cal: calibration; +TV: positive tone of voice (condition); +FE: positive facial expressions; +BM: positive body movements; NC: Neutral control condition. N of Norwegian coders: 15; N of US coders: 6.

**References**

Argyle, M. (2013). *Bodily communication*. Routledge.

Ambady, N., Bernieri, F. J., & Richeson, J. A. (2000). Toward a histology of social behavior: Judgmental accuracy from thin slices of the behavioral stream. In *Advances in experimental social psychology* (Vol. 32, pp. 201-271). Elsevier.

Ekman, P., Friesen, W. V., & Ellsworth, P. (2013). *Emotion in the human face: Guidelines for research and an integration of findings* (Vol. 11). Elsevier.

Friedman, H. S., & Riggio, R. E. (1981). Effect of individual differences in nonverbal expressiveness on transmission of emotion. *Journal of nonverbal behavior*, *6*(2), 96-104.

Zimmerman, L. E. S. (1976). *First impressions as influenced by eye contact, sex and demographic background*. University of Nevada, Reno.

1. Facial expressions [↑](#footnote-ref-1)
2. Body postures and movements [↑](#footnote-ref-2)
3. Tone of voice [↑](#footnote-ref-3)
